# Serum *Helicobacter pylori* FliD antibody and the risk of gastric cancer

**DOI:** 10.18632/oncotarget.7981

**Published:** 2016-03-08

**Authors:** Hailin Li, Bing Zhang, Xiaomeng Hu, Yingzi Dong, Qing Fan, Fang Guo, Xiyun Ren, Haibo Zhou, Wenjing Tian, Yashuang Zhao

**Affiliations:** ^1^ Department of Epidemiology, College of Public Health, Harbin Medical University, Harbin, Heilongjiang Province, P. R. China; ^2^ Xiangfang Center for Disease Control and Prevention, Harbin, Heilongjiang Province, P. R. China

**Keywords:** H. pylori FliD protein, H. pylori CagA protein, serum antibody, gastric cancer, case-control study

## Abstract

FliD and CagA are important virulence factors of *H. pylori*. We aimed to evaluate the screening values of FliD and CagA for gastric cancer (GC). Serum samples were obtained from 232 cases and 266 controls in a case-control study. Unconditional multivariate logistic regression with odds ratios (ORs) and 95% confidence intervals (CIs) was used to analyze the relationships between FliD, CagA and GC. The sensitivities, specificities and receiver operating characteristic (ROC) curves were calculated. Finally, the combined screening values of FliD, FlaA, NapA and CagA were assessed based on discriminant analysis. In all subjects, the associations of FliD and CagA with GC were evident with ORs (95% CIs) of 7.6 (4.7-12.3) and 2.5 (1.6-3.8), respectively (*p<0.001). The areas under ROC curves (AUCs) for FliD and CagA were 0.800 and 0.653, respectively. The AUC for the combination of FliD, FlaA and NapA was 0.915, which represented an increase of 0.115 over that of FliD alone (*p<0.001). These findings indicate that the FliD antibody is associated with GC and could exhibit high validity as a biomarker in screening for GC patients. The combination of FliD, FlaA and NapA improved the screening validity.

## INTRODUCTION

According to GlOBACAN 2012, gastric cancer (GC) is the fifth most common cancer in terms of incidence and the third-leading cause of cancer death worldwide. Moreover, half of the world's GC occurs in Eastern Asia, primarily China [1]. Currently, the reliable methods for identifying subjects who are at a high risk of GC are still limited to gastroscopy and biopsy, but these methods are invasive, expensive and time-consuming [2, 3]. Therefore, noninvasive and acceptable biomarkers for GC are urgently needed.

*Helicobacter pylori (H. pylori)* has been classified as a type-1 carcinogen by the World Health Organization (WHO) and the International Agency for Research on Cancer (IARC) [4]. A meta-analysis of 42 studies demonstrated that *H. pylori* infection is associated with a 2-fold increase in the risk of developing gastric adenocarcinoma [5]. Additionally, another meta-analysis revealed a reduction in the risk of GC following *H. pylori* eradication with a pooled RR of 0.65 [6]. Thus, population-wide screening for and eradication of *H. pylori* infection may be a valid strategy for GC prevention. However, although more than half of the world's population is infected with *H. pylori* [7], the majority of individuals infected with this bacterium are completely asymptomatic and do not demonstrate any severe pathology throughout their lives. Only 1-3% of infected individuals will eventually develop GC [8, 9]. Therefore, it has been postulated that these different outcomes of infection may be partly due to differences in the virulence factors of the infecting *H. pylori* strains [10].

*H. pylori* has a wide variety of immunogenic virulence factors, and the host responses directed against these factors accordingly generate different immune patterns that may be associated with the pathogen-related GC risk to some extent [11]. One study reported that seropositivities for four proteins (i.e., Omp, HP0305, HyuA, and HpaA) are associated with 1.5- to three-fold increases in the risk of GC among Chinese populations [12]. GroES is a dominant GC-related antigen with a much higher seropositivity in GC cases (64.2%) compared with gastritis (30.9%) and duodenal ulcer cases (35.5%) [13]. However, these studies only examined the associations of *H. pylori* virulence factors with GC risk and did not assess the validities of these factors as GC screening markers.

The flagellar hook-associated protein (FliD) is an important *H. pylori* virulence factor that enables flagellin monomers to assemble into a flagella and is thus essential to bacterial motility as demonstrated by infections of mice with a *fliD*-mutant *H. pylori* [14]. Flagella-providing motility further contributes to *H. pylori* colonization and infection [15–17]. Additionally, the FliD protein induces specific antibodies in nearly all infected individuals [18]. Therefore, we performed the present study to assess the association between seropositivity for the antibody against *H. pylori* FliD and the risk of GC and to explore the application of the serum FliD antibody as a novel biomarker for GC. Furthermore, we also studied and assessed the screening value of the antibody against the typical virulence factor cytotoxin-associated gene A (CagA). We further explored the combined screening validity of FliD, CagA, flagellin A (FlaA), and neutrophil-activating protein (NapA), which are proteins that we have previously studied [19, 20].

## RESULTS

### Subject characteristics

The basic demographic and clinical characteristics, behavior, family history of GC and serologic test results for *H. pylori* are presented in Table 1. The differences in the BMI, years of education, smoking, alcohol consumption, *H. pylori* infection and family history of GC distributions between the cases and controls were statistically significance (*p<0.05). Of the 232 patients with GC, only 9 had gastric cardia cancer; 14 (7.2%) of the cases were in stage I, 16 (8.2%) were in stage II, 143 (73.7%) were in stage III, and 21 (10.8%) were in stage IV. The rates of seropositivity for *H. pylori* in the case and control groups were 59.7% and 48.0%, respectively.

**Table 1:** Characteristics of the study subjects

Variables	Case (N=232)		Control (N=266)		P^*^
	N	%	N	%	
Age (years, mean ± SD)	59.6 ± 10.6		57.7 ± 10.6		0.055
Sex					
Male	174	75.0	179	67.3	0.059
Female	58	25.0	87	32.7	
BMI					
<18.5	24	11.8	2	0.8	<0.001
18.5-24.9	135	66.5	174	67.2	
25.0-29.9	36	17.7	76	29.3	
≥30.0	8	3.9	7	2.7	
Years of education					
≤ 8	131	63.9	151	57.9	0.019
9-11	46	22.4	87	33.3	
≥ 12	28	13.7	23	8.8	
Smoking status					
Ever	133	65.5	119	45.6	<0.001
Never	70	34.5	142	54.4	
Alcohol drinking status					
Ever	121	59.3	99	38.1	<0.001
Never	83	40.7	161	61.9	
*H. pylori* serostatus					
Positive	132	59.7	120	48.0	0.011
Negative	89	40.3	130	52.0	
Family history of gastric cancer					
Positive	20	10.1	2	0.8	<0.001
Negative	179	89.9	259	99.2	
Locations					
Non cardia	223	96.1			
Cardia	9	3.9			
Depth of invasion					
T1	17	9.2			
T2	12	6.5			
T3	15	8.2			
T4	140	76.1			
Lymph node metastasis					
N0	68	37.8			
N1	28	15.6			
N2	29	16.1			
N3	55	30.6			
Distant metastasis					
M0	179	89.5			
M1	21	10.5			
Clinical stage					
I	14	7.2			
II	16	8.2			
III	143	73.7			
IV	21	10.8			

* *P* values were obtained from t-tests and chi-square tests.

### Cloning and expression of the recombinant protein

**Figure 1:**
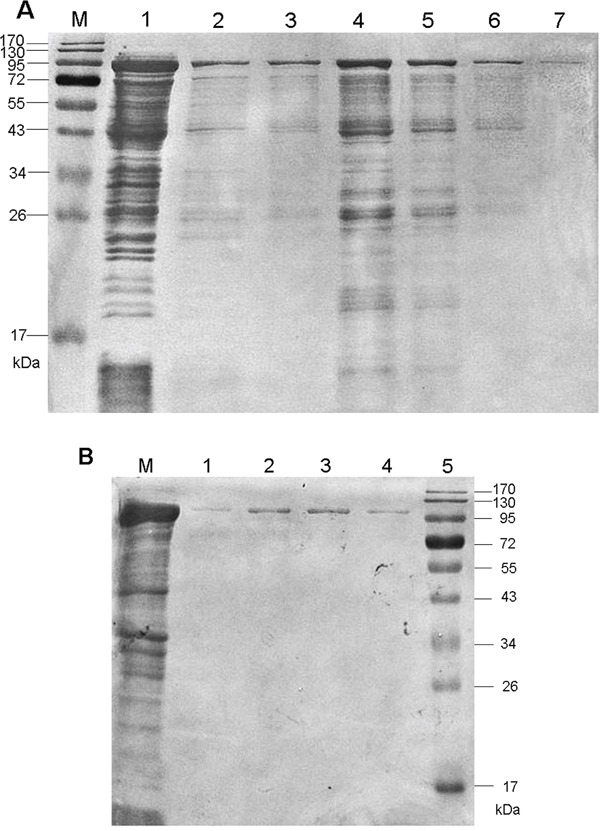
SDS-PAGE analyses of the purified recombinant proteins **A.** FliD. **B.** CagA.

The nucleotide homology of the 2058-bp cloned *fliD* gene relative to *H. pylori* strain J99 was 94.41% (Supplementary Figure S1) [21]. The amplified *cagA* fragment was exactly 2247 bp, and the homology with *H. pylori* strain 26695 was 99.96% (Supplementary Figure S2) [22]. After adding IPTG, bands of rFliD and rCagA with predicted molecular weights of 92 kDa and 103 kDa were found, respectively, in the ultrasonic supernatants and precipitates of the cell lysates by sodium dodecyl sulfate polyacrylamide gel electrophoresis (SDS-PAGE). Finally, the purified soluble recombinant proteins were obtained (Figure 1).

### Association between seropositivity for FliD and CagA antibody and GC

As illustrated in Table 2, the associations between *H. pylori* FliD serum antibody and GC were statistically significant with adjusted odds ratios (ORs) (95%CIs) of 10.6 (5.2-21.6), 6.5 (3.0-14.0), and 7.6 (4.7-12.3) in the *H. pylori*-positive and -negative subjects and the overall subjects (i.e., irrespective of *H. pylori* status), respectively (*p<0.001). Strong, significant dose-response relationships between the serum FliD antibody levels and GC were observed in these three populations (*p<0.001; Table 3).

**Table 2:** Associations of seropositivities for FliD and CagA antibodies with GC in the study subjects

Virulence factor serostatus	*H. pylori*-positive subjects				*H. pylori*-negative subjects				Overall subjects			
	Case N (%)	Control N (%)	OR (95% CI)^b^	*P*	Case N (%)	Control N (%)	OR (95% CI)^b^	*P*	Case N (%)	Control N (%)	OR (95% CI)^b^	*P*
FliD												
Negative	43 (32.6)	99 (82.5)	1.0 (Reference)	<0.001	15 (16.9)	58 (44.6)	1.0 (Reference)	<0.001	49 (21.1)	171 (64.3)	1.0 (Reference)	<0.001
Positive	89 (67.4)	21 (17.5)	10.6 (5.2-21.6)		74 (83.1)	72 (55.4)	6.5 (3.0-14.0)		183 (78.9)	95 (35.7)	7.6 (4.7-12.3)	
CagA												
Negative	24 (18.2)	51 (42.5)	1.0 (Reference)	0.001	24 (27.0)	56 (43.1)	1.0 (Reference)	<0.001	78 (33.6)	150 (56.4)	1.0 (Reference)	<0.001
Positive	108 (81.8)	69 (57.5)	3.0 (1.6-5.9)		65 (73.0)	74 (56.9)	3.8 (1.9-7.7)		154 (66.4)	116 (43.6)	2.5 (1.6-3.8)	

b Adjusted for age, sex, BMI, education, smoking, alcohol consumption and family history of gastric cancer.

**Table 3:** Dose-dependent associations of GC risk with serum FliD and CagA antibody levels in the study subjects

*H*. pylori positive subjects					*H. pylori* negative subjects					overall subjects				
Antibody level (OD)*	Case N (%)	Control N (%)	OR (95% CI)^b^	*P*	Antibody level (OD)*	Case N (%)	Control N (%)	OR (95% CI)^b^	*P*	Antibody level (OD)*	Case N (%)	Control N (%)	OR (95% CI)^b^	*P*
FliD														
> 0.1914	2 (1.5)	30 (25.0)	1.0 (Reference)	<0.001	> 0.1906	2 (2.3)	32 (24.6)	1.0 (Reference)	<0.001	> 0.1841	6 (2.6)	66 (24.8)	1.0 (Reference)	<0.001
0.1313-0.1914	12 (9.1)	30 (25.0)	6.0 (1.4-25.2)		0.1345-0.1906	10 (11.2)	33 (25.4)	4.8 (1.2-19.7)		0.1345-0.1841	19 (8.2)	67 (25.2)	3.1 (1.3-7.7)	
0.1057-0.1313	22 (16.7)	29 (24.2)	11.4 (2.6-49.1)		0.0932-0.1345	26 (29.2)	33 (25.4)	12.6 (3.5-44.7)		0.1028-0.1345	49 (21.1)	67 (25.2)	8.0 (3.5-18.4)	
≤ 0.1057	96 (72.7)	31 (25.8)	46.5 (14.7-147.1)		≤ 0.0932	51 (57.3)	32 (24.6)	25.5 (7.0-93.1)		≤ 0.1028	158 (68.1)	66 (24.8)	26.3(13.7-50.6)	
CagA														
> 0.0544	17 (12.9)	30 (25.0)	1.0 (Reference)	<0.001	> 0.0530	10 (11.2)	31 (23.8)	1.0 (Reference)	<0.001	> 0.0531	29 (12.5)	66 (24.8)	1.0 (Reference)	<0.001
0.0400-0.0544	23 (17.4)	29 (24.2)	1.4 (0.6-3.0)		0.0387-0.0530	10 (11.2)	33 (25.4)	0.9 (0.2-5.0)		0.0393-0.0531	37 (15.9)	63 (23.7)	1.3 (0.8-2.3)	
0.0291-0.0400	33 (25.0)	31 (25.8)	1.9 (0.9-4.1)		0.0270-0.0387	23 (25.9)	33 (25.4)	2.2 (0.9-5.7)		0.0280-0.0393	61 (26.3)	68 (25.6)	2.0 (1.1-3.5)	
≤ 0.0291	59 (44.7)	30 (25.0)	3.5 (1.7-7.4)		≤ 0.0270	46 (51.7)	33 (25.4)	4.3 (1.8-10.3)		≤ 0.0280	105 (45.3)	69 (25.9)	3.5 (2.1-5.8)	

* The Seropositivities for the antibody to FliD were categorized by antibody level quartiles in the controls.

As presented in Table 2, seropositivity for the CagA antibody was also significantly linked to the risk of GC with OR_adjusted_ (95% CI) values of 3.0 (1.6-5.9), 3.8 (1.9–7.7), and 2.5 (1.6–3.8) in the *H. pylori*-positive and -negative subjects and the overall subjects, respectively (*p<0.01). Significant dose-response relationships between the serum CagA antibody levels and GC were also observed (*p<0.001; Table 3).

### Screening utilities of the serum FliD and CagA antibodies for GC

**Figure 2:**
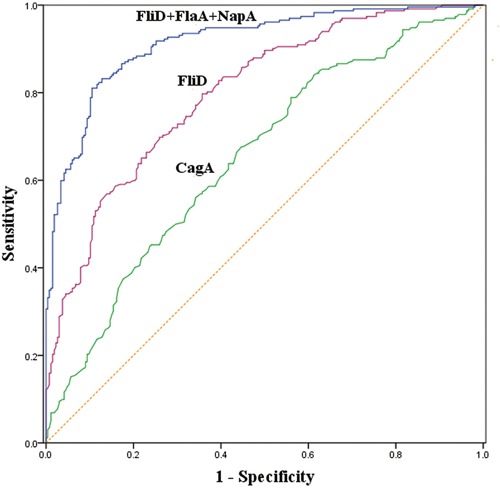
Receiver operating characteristic (ROC) curves of the predictive performances of the serum FliD antibody, CagA antibody and the combination of FliD, FlaA and NapA antibodies in the overall subjects

According an receiver operating characteristic (ROC) analysis of FliD in the *H. pylori*-positive subjects, the optimal FliD cutoff value for GC was 0.1007, which provided a sensitivity of 67.4% and a specificity of 82.5%. The optimal cutoff value was 0.1263 for the *H. pylori*-negative subjects, which resulted in a sensitivity of 83.1% and a specificity of 55.4%. The optimal FliD cutoff value was 0.1153 in the overall group of subjects, and this cutoff generated a sensitivity of 78.9% and a specificity of 64.3%. When the optimal CagA cutoff values were set at 0.0463, 0.0367 and 0.0463 for the *H. pylori*-positive, *H. pylori*-negative and overall subjects, the sensitivities were 81.8%, 73.0% and 83.2%, and the specificities were 42.5%, 56.9% and 39.1%, respectively (Table 4). The areas under ROC curves (AUCs) for FliD and CagA were 0.800 and 0.653, respectively, in the overall groups of subjects (Figure 2).

**Table 4:** Sensitivities and specificities at different FliD and CagA critical values

	*H. pylori* positive subjects			*H. pylori* negative subjects			Overall subjects		
Percentile^a^	critical value (OD)	Sn* (%)	Sp* (%)	critical value (OD)	Sn* (%)	Sp* (%)	critical value (OD)	Sn* (%)	Sp* (%)
FliD									
Optimal cutoff point^b^	0.1007	67.4	82.5	0.1263	83.1	55.4	0.1153	78.9	64.3
25%	0.1057	72.0	75.8	0.0932	57.3	75.4	0.1028	68.1	75.2
50%	0.1313	89.4	50.0	0.1345	86.5	50.0	0.1345	89.2	50.0
75%	0.1914	98.5	25.0	0.1906	97.8	24.6	0.1841	97.4	24.8
90%	0.2694	100.0	10.0	0.2688	98.9	10.0	0.2676	99.6	9.8
CagA									
Optimal cutoff point^b^	0.0463	81.8	42.5	0.0367	73.0	56.9	0.0463	83.2	39.1
25%	0.0291	44.7	75.0	0.0270	50.6	76.9	0.0280	45.3	75.9
50%	0.0400	68.9	50.8	0.0387	77.5	50.8	0.0393	70.7	50.4
75%	0.0544	87.1	25.0	0.0530	88.8	25.4	0.0531	87.5	24.8
90%	0.0696	97.0	10.0	0.0716	96.6	10.0	0.0701	96.6	9.8

a Percentiles of the serum FliD antibody levels in the controls;

* *Sn*, Sensitivity; *Sp*, Specificity.

b The optimal cutoff points for the different biomarkers were based on the maximum Youden's indices (sensitivity + specificity-1).

### Screening utility of the combination of the serum FliD, CagA, FlaA and NapA antibodies for GC

In the present paper, the combined screening validities of FliD, FlaA, NapA and CagA generated by discriminant analysis were also analyzed. As illustrated in Table 5, the AUC and sensitivity of the combination of FliD and FlaA was significantly greater than that of FliD alone. Regarding the combination of FliD, FlaA and NapA, although the AUC was not significantly increased compared to that of the combination of FliD and FlaA, the sensitivity (at the specificity of 90%) was significantly increased by 10.6% in the *H. pylori*-positive subjects. Similarly, in *H. pylori*-negative and overall subjects, the sensitivities (at specificities of 95%) were significantly increased by 15.7% and 9.1%, respectively, relative to the combination of FliD and FlaA. Moreover, the AUCs for the combination of FliD, FlaA, NapA and CagA were not significantly greater than those of the combination of FliD, FlaA and NapA among the three populations mentioned above.

**Table 5:** AUCs and sensitivities of the combination of FliD, FlaA, NapA and CagA for GC

Marker combination	AUC (95% CI)	*p*-value for comparison of AUC to FliD	90% Specificity (%)	95% Specificity (%)	98% Specificity (%)	*p*-value for comparison of sensitivity at 95% specificity to FliD
*H. pylori*-positive subjects						
FliD	0.821 (0.768-0.867)	N/A	56.1	39.4	22.7	N/A
FliD+ FlaA	0.913 (0.871-0.945)	<0.001	66.7	60.6	50.0	<0.001
FliD+ NapA	0.822 (0.769-0.867)	0.657	56.1	40.9	25.0	0.617
FliD+ FlaA+NapA	0.918 (0.877-0.949)	<0.001	77.3	61.4	53.0	<0.001
FliD+ FlaA+NapA+CagA	0.916 (0.875-0.947)	<0.001	72.0	62.9	51.5	<0.001
*H. pylori*-negative subjects						
FliD	0.764 (0.702-0.818)	N/A	30.3	25.8	14.6	N/A
FliD+ FlaA	0.891 (0.842-0.929)	<0.001	61.8	36.0	24.7	0.164
FliD+ NapA	0.791 (0.731-0.843)	0.094	42.7	22.5	14.6	0.579
FliD+ FlaA+NapA	0.897 (0.849-0.934)	<0.001	60.7	51.7	29.2	<0.001
FliD+ FlaA+NapA+CagA	0.908 (0.862-0.943)	<0.001	65.2	47.2	22.5	0.004
Overall subjects						
FliD	0.800 (0.762-0.834)	N/A	32.8	21.1	12.5	N/A
FliD+ FlaA	0.907 (0.878-0.931)	<0.001	54.3	43.1	31.9	<0.001
FliD+ NapA	0.805 (0.767-0.839)	0.278	33.6	22.4	14.7	0.450
FliD+ FlaA+NapA	0.915 (0.887-0.938)	<0.001	61.6	52.2	33.2	<0.001
FliD+ FlaA+NapA+CagA	0.915 (0.887-0.938)	<0.001	63.8	44.0	27.2	<0.001

## DISCUSSION

Currently, most countries lack national strategies or recommendations for early GC detection because no good screening methods are available. Hence, screening tests that can reliably evaluate GC subjects are urgently needed.

It has been proposed that the colonization of the gastric mucosa is a prerequisite for *H. pylori* infection and carcinogenesis and that motility is also essential for the initial colonization [17, 23, 24]. Flagella have been generally regarded as important virulence factors of pathogenic bacteria primarily because of the associated motility properties [25]. The FliD protein is thought to function as a capping structure at the distal end of the filament to assemble into a flagella [26, 27]. To our knowledge, only a single study, Kimmel et al. has examined the association between the FliD antibody and GC, and no association was found [28]. In the present study, seropositivity for the FliD antibody was associated with a 10.6-fold increase in the risk of GC. Our result is not in line with that of Kimmel et al., which may be attributable to the small sample size in the latter study (4 cases and 5 controls) or the existing diversity of *H. pylori* and genetic differences in host immune responses to *H. pylori* infection.

In the present study, a commercial ELISA method was used to detect the *H. pylori* infection statuses of the subjects. However, some studies have indicated that commercial ELISA serology may fail to detect past *H. pylori* infections and that patients with negative *H. pylori* serologic tests can actually be positive for anti-CagA antibodies [29, 30]. At this point, the associations between GC and seropositivity for the FliD antibody were also analyzed in the *H. pylori*-negative and overall subjects. Additionally, strong, significant dose-response relationships between serum FliD antibody levels and GC were observed. These data suggest that attention should be focused on the antibody titer in addition to seropositivity.

Although CagA is an important and best-studied virulence factor of *H. pylori*, the association between the CagA antibody and the risk of GC remains controversial [31]. In the present study, we cloned a relatively conserved 2247-bp fragment of *cagA* extending from the 67^th^ to the 2313^th^ bp to express the CagA protein because the fragments of approximately 65 bp starting from the 5′-end of the *cagA* genes of different *H. pylori* isolates exhibit frequent mutations, and a variable EPIYA region at the C-terminus and an important cleavage site preceding the first EPIYA motif also exist [32–34]. The OR for the risk of GC based on the anti-CagA antibody was higher in the *H. pylori*-negative population than in the *H. pylori*-positive and total populations. Our result is consistent with the result of a meta-analysis that suggested that *H. pylori*-negative/CagA-positive subjects may represent the group with the highest risk for GC [31]. However, the AUC for the CagA antibody in all subjects was 0.653. AUCs in the range of 0.97 and above are generally considered to have excellent accuracies, AUCs of 0.93 to 0.96 are considered very good, AUCs of 0.75 to 0.92 are considered good, and AUCs below 0.75 should be cautiously evaluated because the associated tests have obvious deficiencies in accuracy that are approaching random [35, 36]. According to these standard, the accuracy of CagA in the screening of the GC population in our study was poor, and this result is consistent with the conclusion of a meta-analysis that indicated that the CagA antibody should not be used as a GC marker in East Asian countries due to its AUC of 0.636 [37]. Therefore, we focused on other potentially carcinogenic *H. pylori* virulence factors including FliD.

Although gastroscopy and biopsy are reliable methods for GC detection, these procedures are invasive, time-consuming and expensive; therefore, it is necessary to search for ideal biomarkers of GC. Many conventional biomarkers, such as the carcinoembryonic antigen (CEA), cancer antigen 19-9 (CA19-9), and cancer antigen 72-4 (CA72-4), are widely utilized in the clinic, but low sensitivities or low specificities reduce the clinical practical value of these markers for the early diagnosis of GC [38–40]. With the increase quantity of in-depth research into biomarkers for GC, increasing attention has been focused on the use of circulating nucleic acids as novel biomarkers. However, DNA/RNA extraction and bisulfite conversion are too time-intensive for clinical use [41–43]. Therefore, additional studies should be performed to identify the most promising markers for the potential future screening of GC. Emerging evidence has revealed that many *H. pylori* virulence factors promote the development of GC [44]. Additionally, seropositivities for antibodies against some of these *H. pylori* virulence factors have been identified as biomarkers of GC [45, 46]. The *H. pylori* flagellar virulence factor FliD has been identified as an efficient tool for the detection of *H. pylori* infections via measurements of the levels of the antibody to this factor in the serum [18]. In the present study, we explored the value of the serum FliD antibody as a biomarker for distinguishing GC patients from controls. Additionally, the significance of combinations of the FliD, FlaA, NapA and CagA antibodies were assessed. We found that FliD may be an independent biomarker based on the associated AUC of 0.800 in the overall group subjects and that the combination of FliD, FlaA and NapA exhibited a higher validity in the detection of GC with the AUC of 0.915.

Overall, the FliD antibody may be an independent biomarker for GC patients, and the combination of FliD, FlaA and NapA performed even better. However, our findings were obtained in a case-control study, and additional larger prospective studies are needed to expand and confirm these findings.

## MATERIALS AND METHODS

### Study subjects

A hospital-based case-control study was performed in Harbin, Heilongjiang Province, China. All 232 cases with pathologically confirmed GC were enrolled from the Third Affiliated Hospital of Harbin Medical University between March and June of 2010. Additionally, 183 healthy individuals who underwent physical examinations at the Center for Disease Control of Xiangfang District, Harbin between April and July of 2010 and 83 cancer-free neurological patients of the Fourth Affiliated Hospital of Harbin Medical University between March and May of 2011 were included as controls. All participants completed a face-to-face questionnaire and provided written informed consent. A blood sample was drawn from each consenting participant according to a research protocol approved by the Human Research and Ethics Committee of Harbin Medical University. All blood samples were centrifuged and stored at −80°C until use.

### Serologic tests for *H. pylori* by ELISA

*H. pylori* IgG antibodies were measured in duplicate using an enzyme immunoassay kit (IBL, Germany). The sensitivity and specificity of the kit were both greater than 95% according to the manufacturer.

### Recombinant protein cloning and expression

The genomic DNA of a clinical strain provisionally named HLJ014a was extracted using a DNA extraction kit (Qiagen, USA) and stored at −80°C. Briefly, the full-length fliD gene was amplified by polymerase chain reaction (PCR) using the genomic DNA as the template. Oligonucleotide primers were designed based on the published literature [18]. Subsequently, EcoRI sites were introduced into the 5′ ends of the forward primers, and XhoI sites were inserted into the 5′ ends of the reverse primers. The target DNA amplification product was cloned into the *pMD19T (simple)* cloning vector and then transformed into *E. coli* strain DH5α. The positive recombinants were confirmed by bacterial solution PCR, restriction enzyme digestion and DNA sequencing. The digested gel-purified *fliD* gene was ligated into the expression vector *pET-32a(+)*. The recombinant plasmid *pET32a-fliD* was transformed into *E. coli* BL21DE3 cells, transformation was confirmed, and the cells were inoculated into LB medium with 100 μg/mL ampicillin. Expression was induced via the addition of IPTG at a final concentration of 1.0 mmol/L at 30°C and an optical density (OD) of 0.6-0.8. The *E. coli* cells were harvested after 4 hours and lysed via ultrasonication. The suspension was collected and examined with 15% SDS-PAGE. The soluble histidine-tagged protein was purified with Ni-NTA His Bind resin (Novagen, Germany).

We also used the method described above to obtain the purified CagA protein. For the PCR, the primers were designed to amplify a relatively conserved 2247-bp *cagA* fragment extending from the 67^th^ to the 2313^th^ bp at the 5′ end of the *cagA* gene of *H. pylori* strain 26695. IPTG was added to the LB-Amp broth at 30°C to induce CagA protein expression.

### Enzyme-linked immunosorbent assay (ELISA)

The presences of serum IgG antibodies to the recombinant FliD and CagA were determined by indirect ELISA. Ninety-six-well plates (Costar, USA) were coated with 100 μL/well of purified FliD or CagA protein diluted to 0.5 μg/mL and 0.25 μg/mL, respectively, and incubated overnight at 4°C. After washing three times with 300 μL phosphate-buffered saline tween (PBST) (0.15 mol/L phosphate buffer, 0.05% Tween-20, pH 7.4), the ELISA plates were blocked in a moist chamber with 200 μL PBS containing 1% bovine serum albumin (BSA) (Amresco, USA) per well for 2 hours at 37°C. All serologic samples (3600-fold diluted with 0.1% BSA, phosphate buffer) were then added to the microtiter wells and incubated at 37°C for 1 hour. After three washes, 1:10000 or 1:5000 of 100 μL peroxidase-conjugated goat anti-human IgG (ZSGB-Bio, Beijing, China) were loaded into each well for FliD and CagA, respectively, and the plates were incubated for 30 minutes at 37°C. The plates were washed, and TMB substrate (100 μL) was then added to the plates, which were then incubated at 37°C for 15 minutes. The reaction was terminated with the addition of 50 μL of 2 M sulfuric acid solution. Finally, the density of each serum sample was measured at the dual wavelengths of 450 and 630 nm in a microplate reader (BioTek Synergy 2, USA). Each serum sample was tested in triplicate. Additionally, 100 μL PBS containing 0.1% BSA was added to the wells as a control.

### Statistical analysis

We used the t-test to compare the means of the continuous variables between the patients and controls, and the chi-square test was used to compare the distributions of the various characteristics between the different groups. The ELISA values for FliD and CagA were tested with the K-S test following logarithmic conversion. The ELISA result for a patient's serum sample was considered positive if the absorbance (OD450) was less than or equal to the optimal cutoff OD value. Unconditional logistic-regression analysis was conducted to assess the associations between FliD, CagA and GC. To determine the relative risks, ORs and 95% confidence intervals (CIs) were calculated. The chi-square trend test was used to measure the dose-response relationships between the serum FliD and CagA antibody levels and GC. The sensitivities, specificities, and AUCs with the 95% CIs were also calculated. Furthermore, the ELISA values for FliD, FlaA, NapA and CagA were combined with discriminant analysis through the predicted probability for each subject. The AUCs of the combinations were also computed. A test result was considered positive if it was less than or equal to a selected predicted probability and negative if it was greater than a selected predicted probability. According to the selected predicted probability, the sensitivities were obtained at set specificities of 90%, 95%, and 98%. All of the above-mentioned statistical analyses were performed with SPSS statistics 17.0, and with *P* values <0.05 were considered statistically significant.

## SUPPLEMENTARY MATERIALS TABLES AND FIGURES







## Abbreviations

FliD: flagellar hook-associated protein
CagA: cytotoxin-associated gene A
FlaA: flagellin A
NapA: neutrophil-activating protein
GC: gastric cancer
*H. pylori*: *Helicobacter pylori*
WHO: World Health Organization
IARC: International Agency for Research on Cancer
PCR: polymerase chain reaction
CAG: chronic atrophy gastritis
ELISA: enzyme-linked immunosorbent assay
OD: optical density
OR: odds ratio
CI: confidence interval
ROC: receiver operating characteristic
AUC: area under the ROC curve
SDS-PAGE: sodium dodecyl sulfate polyacrylamide gel electrophoresis
PBST: phosphate-buffered saline tween
BSA: bovine serum albumin
CEA: carcinoembryonic antigen
CA19-9: cancer antigen 19-9
CA72-4: cancer antigen 72-4
