# Supplementary material for: Serum Helicobacter pylori FliD antibody and the risk of gastric cancer
Source: Oncotarget. 2016 Mar 8;7(16):22397–408. doi: 10.18632/oncotarget.7981 (PMC5008368; doi:10.18632/oncotarget.7981)
Supplement: Supplementary file 1 [file oncotarget-07-22397-s001.pdf]

Serum *Helicobacter pylori* FliD antibody and the risk of gastric cancer

SUPPLEMENTARY MATERIALS

Supplementary Figure S1: Sequence alignment of the cloned *fliD* gene with corresponding *fliD* gene of *H. pylori* reference strain J99. A. the cloned *fliD* gene. **B.** the *fliD* gene of *H. pylori* reference strain J99.

See Supplementary File 1

Supplementary Figure S2: Sequence alignment of the cloned *cagA* gene with corresponding *cagA* gene of *H. pylori* reference strain 26695. A. the cloned *cagA* gene. **B.** the *cagA* gene of *H. pylori* reference strain 26695.

See Supplementary File 2
