# Supplementary material for: Serum Helicobacter pylori FliD antibody and the risk of gastric cancer
Source: Oncotarget. 2016 Mar 8;7(16):22397–408. doi: 10.18632/oncotarget.7981 (PMC5008368; doi:10.18632/oncotarget.7981)
Supplement: Supplementary file 2 [file oncotarget-07-22397-s002.doc]

**Serum *Helicobacter pylori* FliD antibody and the risk of gastric cancer**

**Supplementary Materials**

(A) 1 ATGGCAATAGGTTCATTAAGCTCATTAGGGCTTGGCAGTAAGGTTTTGAATTACGATGTG

(B) 1 ............................................................

(A) 61 ATTGACAAGCTTAAGGACGCTGATGAAAAAGCGTTAATCGCCCCCTTAGACAAGAAAATG

(B) 61 ................................T...........AC..............

(A) 121 GAGCAAAATGTTGAAAAACAAAAAGCCCTTGTGGAAATTAAAACGCTCCTTTCTTCTCTC

(B) 121 .....................................................A.....A

(A) 181 AAAGGCCCGGTTAAAACGCTTTCAGATTATTCCACTTATATCAGCAGGAAAAGCAATGTT

(B) 181 ....................C........................C.A............

(A) 241 ACAGGCGATGCGTTGAGCGCGAGCGTGGGGGCTGGCGTGCCTATTCAAGATATTAAAGTG

(B) 241 ............................................C...........G...

(A) 301 GATGTGCAAAATTTAGCGCAAGGCGATATTAACGAATTAGGGGCGAAATTTTCTTCAAGA

(B) 301 ............................................................

(A) 361 GATGATATTTTTAGCCAAGTGGATACCACGCTCAAGTTTTACACGCAAAACAAGGACTAC

(B) 361 .............................A..........................T...

(A) 421 GCCGTTAACATTAAAGCAGGAATGACTTTAGGCGATGTGGCTCAAAGCATCACGGACGCT

(B) 421 ......G.T...................................................

(A) 481 ACCAACGGCGAAGTGATGGGCATTGTGATGAAAACAGGAGGGAATGACCCCTACCAATTA

(B) 481 .....T......................................................

(A) 541 ATGGTGAATACCAAAAACACCGGTGAAGACAACCACATTTATTTTGGCTCACACCTCCAA

(B) 541 .......................C..........GGG.C.....................

(A) 601 TCCACGCTCACCAACAAAAACGCCCTTTCTTTGGGGGTTGATGGAGCCGGAAAAAGTGAA

(B) 601 ...........T....................A..AC.............G.....C...

(A) 661 GTGAGTTTGAATTTAAAGGGGGCTGATGGGAGTATGCATGAAGTCCCCATTGTGCTAGAA

(B) 661 T...............................C..............T..CA.......G

(A) 721 CTCCCTGAAAGCGCTTCTATCAAACAAAAAAACACCGCAATCCAAAAAGCGATAGAGCAG

(B) 721 .................................G.A..G........G..T..G......

(A) 781 GCTTTAGAAAATGACCCTAATTTTAAAGATTTGATCGCTAATGGGGATATTTCTATAGAC

(B) 781 ...........C.................C.......................C......

(A) 841 ACTCTTCATGGGGGGGAGTCTTTAATCATTAATGACAGGCGTGGGGGAAATATTGAAGTT

(B) 841 .................A..........................A.....C.........

(A) 901 AAAGGGAGTAAGGCTAAAGAGCTTGGGTTTTTGCAAACCACCACCCAAGAAAGCGATTTA

(B) 901 ...............................C...........................G

(A) 961 TTGAAAAGCTCTCGCACCATAAAAGAGGGTAAATTAGAAGGGGCGATTAGCTTGAATGGC

(B) 961 ..A......G.......G.........................T.G....T.........

(A) 1021 CAAAAACTGGATTTGAGCGCTTTAACCAAAGAAAGCAACACCAGTGAAGAAAACACAGAC

(B) 1021 ......T.............C...........G...........C...............

(A) 1081 GCTATCATTCAAGCGATCAATTCCAAAGAAGGCTTGAATGCGTTTAAGAACGCCGAAGGC

(B) 1081 ....................CG.T....................................

(A) 1141 AAGCTTGTGATCAATTCTAAAACCGGGATGCTAACGATTAAGGGCGAGGACGCTTTAGGT

(B) 1141 ..........................A.....C..C.......................C

(A) 1201 AAGGCCAGTTTGAAGGATTTGGGCTTGAATGCTGGCATGGTGCAATCCTATGAAGCTTCA

(B) 1201 .....T.................T....GC.................T...........G

(A) 1261 CAAAACACGCTTTTTATGTCTAAAAATTTGCAAAAAGCGAGCGATTCAGCATTCACTTAT

(B) 1261 ...G...................G.....A.................GCA.........C

(A) 1321 AACGGGGTGAGCATCACACGCCCCACTAATGAGGTCAATGATGTGATCAGCGGGGTTAAT

(B) 1321 ..T..............G..........................................

(A) 1381 ATCACTTTAGAGCAAACCACAGAGCCTAATAAACCTGCCATTATCAGCGTGAGCAGGGAC

(B) 1381 ................................G.......................A...

(A) 1441 AATCAAGCCATTATAGACAGCCTTACTGAATTTGTGAAAGCCTATAATGAGCTTATCCCC

(B) 1441 .........................AA........C.......................T

(A) 1501 AAATTAGATGAAGACACTCGTTATGACGCTGACACTAAAATCGCTGGGATTTTTAACGGC

(B) 1501 ...C....C........G..C.......................C...............

(A) 1561 GTGGGCGATATTCGCGCGATTCGTTCTTCTCTTAATAATGTGTTTTCTTATAGCGTGCAT

(B) 1561 ..............T..C...A.A..C.................................

(A) 1621 ACGGATAATGGGGTAGAAAGCTTGATGAAATACGGGCTTAGTTTGGACGATAAAGGCGTG

(B) 1621 ............................................A........G......

(A) 1681 ATGAGTTTAGATGAGGCTAAATTGAGTAGTGCCTTAAATTCTAATCCTAAAGCGACTCAA

(B) 1681 ........G.....A........ATCA.....A...........C...............

(A) 1741 GATTTTTTCTATGGGAGCGATAGCAAGGATATGGGGGGCAGAGAAATCCACCAAGAGGGC

(B) 1741 ............................................................

(A) 1801 ATTTTTTCTAAATTCAATCAAGTCATCGCCAATCTCATAGATGGAGGGAACGCTAAATTA

(B) 1801 .............................T..C...........................

(A) 1861 AAGATTTATGAAGATTCCCTAGACAGAGACGCTAAAAGCCTGACCAAAGACAAAGAAAAC

(B) 1861 ............................................................

(A) 1921 GCTCAAGAGCTTTTAAAAACCCGCTACAACATCATGGCGGAACGCTTTGCGGCTTATGAC

(B) 1921 ..........................T..............G........C........T

(A) 1981 AGCCAAATTTCTAAAGCCAATCAAAAATTCAATTCCGTGCAAATGATGATCGATCAAGCA

(B) 1981 ..T.....C...................................................

(A) 2041 GCGGCTAAAAAGAATTAA

(B) 2041 ..................

**Supplementary figure 1: Sequence alignment of the cloned *fliD* gene with corresponding *fliD* gene of *H. pylori* reference strain J99.** (A) the cloned *fliD* gene. (B) the *fliD* gene of *H. pylori* reference strain J99.
