# Supplementary material for: Serum Helicobacter pylori FliD antibody and the risk of gastric cancer
Source: Oncotarget. 2016 Mar 8;7(16):22397–408. doi: 10.18632/oncotarget.7981 (PMC5008368; doi:10.18632/oncotarget.7981)
Supplement: Supplementary file 3 [file oncotarget-07-22397-s003.doc]

(A) 1 CCGCAACAATTTATCAATAATCTTCAAGTGGCTTTTATTAAAGTTGATAATGTTGTCGCT

(B) 1 ............................................................

(A) 61 TCATTTGATCCTGATCAAAAACCAATCGTTGATAAGAACGATAGGGATAACAGGCAAGCT

(B) 61 ............................................................

(A) 121 TTTGATGGAATCTCGCAATTAAGGGAAGAATACTCCAATAAAGCGATCAAAAATCCTACC

(B) 121 ............................................................

(A) 181 AAAAAGAATCAGTATTTTTCAGACTTTATCGATAAGAGCAATGATTTAATCAACAAAGAC

(B) 181 ............................................................

(A) 241 AATCTCATTGATGTAGAATCTTCCACAAAGAGCTTTCAGAAATTTGGGGATCAGCGTTAC

(B) 241 ............................................................

(A) 301 CAAATTTTCACAAGTTGGGTGTCCCATCAAAAAGATCCGTCTAAAATCAACACCCGATCG

(B) 301 ............................................................

(A) 361 ATCCGAAATTTTATGGAAAATATCATACAACCCCCTATCCCTGATGATAAAGAAAAAGCA

(B) 361 ............................................................

(A) 421 GAGTTTTTGAAATCTGCCAAACAATCTTTTGCAGGAATCATTATAGGGAATCAAATCCGA

(B) 421 ............................................................

(A) 481 ACGGATCAAAAGTTCATGGGCGTGTTTGATGAATCCTTGAAAGAAAGGCAAGAAGCAGAA

(B) 481 ............................................................

(A) 541 AAAAATGGAGGGCCTACTGGTGGGGATTGGTTGGATATTTTTCTCTCATTTATATTTAAC

(B) 541 ............................................................

(A) 601 AAAAAACAATCTTCCGATGTCAAAGAAGCAATCAATCAAGAACCAGTTCCCCATGTCCAA

(B) 601 ............................................................

(A) 661 CCAGATATAGCCACTACTACCACCGACATACAAGGCTTACCGCCTGAAGCTAGGGATTTA

(B) 661 ............................................................

(A) 721 CTTGATGAAAGGGGTAATTTTTCTAAATTCACTCTTGGCGATATGGAAATGTTAGATGTT

(B) 721 ............................................................

(A) 781 GAGGGAGTCGCTGACATTGATCCTAATTACAAGTTCAATCAATTATTGATTCACAATAAC

(B) 781 ............................................................

(A) 841 GCTCTGTCTTCTGTGTTAATGGGGAGTCATAATGGCATAGAACCTGAAAAAGTTTCATTA

(B) 841 ............................................................

(A) 901 TTGTATGCGGGCAATGGTGGTTTTGGAGACAAACACGATTGGAACGCCACCGTTGGTTAT

(B) 901 ............................................................

(A) 961 AAAGACCAACAAGGTAACAATGTGGCTACACTCATTAATGTGCATATGAAAAACGGCAGT

(B) 961 ............................................................

(A) 1021 GGCTTAGTCATAGCAGGTGGTGAGAAAGGGATTAATAACCCTAGTTTTTATCTCTACAAA

(B) 1021 ............................................................

(A) 1081 GAAGACCAACTCACAGGCTCACAACGAGCATTGAGTCAAGAAGAGATCCGAAACAAAGTA

(B) 1081 ............................................................

(A) 1141 GATTTCATGGAATTTCTTGCACAAAATAATACTAAATTAGACAACTTGAGCGAGAAAGAG

(B) 1141 ............................................................

(A) 1201 AAAGAAAAATTCCAAAATGAGATTGAAGATTTTCAAAAAGACTCTAAGGCTTATTTAGAC

(B) 1201 ............................................................

(A) 1261 GCCCTAGGGAATGATCGTATTGCTTTTGTTTCTAAAAAAGACACAAAACATTCAGCTTTA

(B) 1261 ............................................................

(A) 1321 ATTACTGAGTTTAATAATGGGGATTTGAGCTACACTCTCAAAGATTATGGGAAAAAAGCA

(B) 1321 ............................................................

(A) 1381 GATAAAGCTTTAGATAGGGAGAAAAATGTTACTCTTCAAGGTAGCCTAAAACATGATGGC

(B) 1381 ............................................................

(A) 1441 GTGATGTTTGTTGATTATTCTAATTTCAAATACACCAACGCCTCCAAGAATCCCAATAAG

(B) 1441 ............................................................

(A) 1501 GGTGTAGGCGCTACGAATGGCGTTTCCCATTTAGAAGCAGGCTTTAACAAGGTAGCTGTC

(B) 1501 ............................................................

(A) 1561 TTTAATTTGCCTGATTTAAATAATCTCGCTATCACTAGTTTCGTAAGGCGGAATTTAGAG

(B) 1561 ............................................................

(A) 1621 AATAAACTAACCGCTAAAGGATTGTCCCTACAAGAAGCTAATAAGCTCATCAAAGACTTT

(B) 1621 ............................................................

(A) 1681 TTGAGCAGCAACAAAGAATTGGCTGGAAAAGCTTTAAACTTCAATAAAGCTGTAGCTGAA

(B) 1681 ............................................................

(A) 1741 GCTAAAAACACAGGCAACTATGACGAGGTGAAAAAAGCTCAGAAAGATCTTGAAAAATCC

(B) 1741 .......G....................................................

(A) 1801 CTAAGGAAACGAGAGCATTTGGAGAAAGAAGTAGAGAAAAAATTGGAGAGCAAAAGCGGC

(B) 1801 ............................................................

(A) 1861 AACAAAAATAAAATGGAAGCAAAAGCTCAAGCTAACAGCCAAAAAGATGAGATTTTTGCG

(B) 1861 ............................................................

(A) 1921 TTGATCAATAAAGAGGCTAATAGGGATGCAAGAGCAATCGCTTACACTCAAAATCTTAAA

(B) 1921 ............................................................

(A) 1981 GGCATCAAAAGGGAATTGTCTGATAAACTTGAAAAAATCAGCAAGGATTTGAAAGACTTT

(B) 1981 ............................................................

(A) 2041 AGTAAATCTTTTGATGAATTCAAAAATGGCAAAAATAAGGATTTCAGCAAGGCAGAAGAA

(B) 2041 ............................................................

(A) 2101 ACGCTAAAAGCCCTTAAAGGCTCGGTGAAAGATTTAGGTATCAATCCAGAATGGATTTCA

(B) 2101 ............................................................

(A) 2161 AAAGTTGAAAACCTTAATGCAGCTTTGAATGAATTCAAAAATGGCAAAAATAAGGATTTC

(B) 2161 ............................................................

(A) 2221 AGCAAGGTAACGCAAGCAAAAAGCGAC

(B) 2221 ...........................

**Supplementary figure 2: Sequence alignment of the cloned *cagA* gene with corresponding *cagA* gene of *H. pylori* reference strain 26695.** (A) the cloned *cagA* gene. (B) the *cagA* gene of *H. pylori* reference strain 26695.
